# Primary Lymphoma of Peripheral Nerve: Rare or Misdiagnosed? A Systematic Review

**DOI:** 10.3390/life15091357

**Published:** 2025-08-27

**Authors:** Ludovico Caruso, Adriano Cannella, Giulia Maria Sassara, Antonio Maria Rapisarda, Marco Passiatore, Giuseppe Rovere, Rocco De Vitis

**Affiliations:** 1 Department of Ageing, Neurosciences, Head-Neck and Orthopaedics Sciences, Hand Surgery Unit, Fondazione Policlinico Universitario Agostino Gemelli IRCSS, 00168 Rome, Italy; ludivicocaluso.lc@gmail.com (L.C.); adriano.cansa@gmail.com (A.C.); giuliamariasassara@gmail.com (G.M.S.); antoniomariarapisarda@gmail.com (A.M.R.); 2 Department of Orthopedic and Geriatric Sciences, Catholic University of the Sacred Heart, 00168 Rome, Italy; 3 Department of Orthopaedics, Azienda Ospedaliera San Giovanni Addolorata, 00184 Rome, Italy; 4 Department of Clinical Science and Translational Medicine, Section of Orthopaedics and Traumatology, University of Rome “Tor Vergata”, 00133 Rome, Italy; giuseppe.rovere02@icatt.it; 5 Unit of Orthopaedics, “Spedali Civili” Hospital, University of Brescia, 25123 Brescia, Italy; passiatore.m@gmail.com

**Keywords:** tumors, peripheral nerve, primary lymphoma

## Abstract

Background: Primary lymphoma of peripheral nerves (PLPN) is a rare extranodal non-Hodgkin lymphoma that mimics benign nerve conditions, leading to diagnostic delays. This systematic review evaluates the clinical, radiological, and pathological features of PLPN, alongside diagnostic and therapeutic strategies. Materials and Methods: A systematic search was conducted across PubMed, Scopus, and Web of Science, and identified 23 studies reporting 27 cases of PLPN. Data on demographics, clinical presentation, diagnostics, treatment, and outcomes were extracted and synthesized qualitatively due to study heterogeneity. Results: The sciatic nerve was most involved (48.15%), followed by the ulnar (18.5%) and radial nerves (18.5%). The median age at diagnosis was 58 years, with symptoms including motor deficits (88.9%), sensory disturbances (74.1%), and pain (70.4%). B-cell lymphomas accounted for 81.5% of cases, predominantly diffuse large B-cell lymphoma. MRI findings were non-specific; however, diffusion-weighted imaging (DWI) showed diagnostic potential. Treatments included combination therapies (51.9%), chemotherapy (25.9%), and surgery. Complete remission was achieved in 70.8%, with a 2-year survival rate of 83.3%. Conclusions: PLPN is rare but likely underdiagnosed. Early recognition requires multidisciplinary collaboration, advanced imaging, and standardized protocols. Future research should focus on molecular characterization, diagnostic criteria, and treatment optimization to improve outcomes for this challenging condition.

## 1. Introduction

Peripheral nerve involvement in lymphoma is a complex and multifaceted clinical scenario that presents significant diagnostic challenges. Approximately 5% of lymphoma patients experience peripheral nervous system (PNS) manifestations, which may result from various mechanisms including direct compression by lymphomatous masses, the infiltration of lymphoma cells into nerve structures, or indirect damage through immune-mediated, metabolic, toxic, or infectious processes. Therapy-related neurotoxicity may also contribute to neurological symptoms in these patients [1,2].

Among these manifestations is a distinct clinical entity known as neurolymphomatosis (NL), which is characterized by the direct invasion of nerve trunks, roots, plexuses, or cranial nerves by lymphomatous cells. When NL represents the initial manifestation of a hematological malignancy, it is classified as primary NL [2,3,4,5]. Within this category, a subset of cases where lymphoma cells are exclusively found in the peripheral nervous system, without evidence of systemic dissemination, is termed primary lymphoma of peripheral nerves (PLPN) [4,5]. This entity stands in contrast to peripheral nerve involvement as a manifestation of systemic disease spread [2,3].

PLPN represents an extraordinarily rare subtype of extranodal non-Hodgkin lymphoma, with fewer than 100 well-documented cases in the literature in upper or lower limbs [5,6,7,8,9,10,11,12,13,14,15,16,17,18,19,20,21,22,23,24,25,26,27,28]. Notably, the sciatic nerve appears to be involved in more than half of the reported cases [6,7,8,9,10,11,12,13,14,15,16]. The rarity of PLPN, combined with its variable clinical presentation and non-specific imaging findings, creates substantial diagnostic challenges. Currently, no standardized diagnostic protocols or treatment guidelines exist, potentially leading to delayed diagnosis and suboptimal management.

The clinical presentation of PLPN can be multifocal [5], but often mimics more common conditions, including compressive neuropathies and benign peripheral nerve sheath tumors such as schwannomas or neurofibromas [6,7,8,9,10,11,12,13,14,15,16,17,18,19,20,21,22,23,24,25,26,27,28]. This similarity to benign conditions, coupled with the absence of systemic disease manifestations, frequently leads to misdiagnosis and inappropriate initial management. The symptoms typically develop unilaterally and asymmetrically, with a relatively rapid progression that can provide subtle clues to its malignant nature.

PLPN exhibits a heterogeneous clinical course, with some cases resembling benign neuropathies due to gradual symptom progression, while others demonstrate a more aggressive trajectory, as evidenced by the rapid worsening of symptoms observed in 78% of cases, leading to significant disability within a median of 3.8 months.

The immunopathological mechanisms underlying PLPN remain poorly understood, with the current hypotheses suggesting lymphocyte homing receptors, nerve-specific microenvironmental factors, and disruption of the blood–nerve barrier as potential causes [2]. Understanding these mechanisms is crucial for developing targeted diagnostic and therapeutic approaches.

While traditional concepts have emphasized the blood–nerve barrier as a protective mechanism against lymphomatous infiltration, emerging evidence suggests that certain lymphoma subtypes may possess unique neurotropic properties that facilitate invasion of peripheral nerve tissue. Recent genomic analyses have identified potential molecular signatures associated with neurotropism in lymphoid malignancies, though these findings require further validation in PLPN-specific contexts [2,3].

Given the diagnostic complexity and potentially aggressive clinical course of PLPN, this systematic review aims to comprehensively analyze the existing literature on primary lymphoma of peripheral nerves, characterizing the clinical, radiological, and pathological features that may facilitate earlier diagnosis, evaluating current diagnostic and therapeutic approaches, and assessing outcomes and prognostic factors

To determine whether PLPN is truly rare, or simply underdiagnosed due to its clinical and radiological similarities to more common conditions, could enhance awareness of this entity and provide evidence-based recommendations for its diagnosis and management.

## 2. Materials and Methods

### 2.1. Search Strategy

A systematic literature search was conducted following PRISMA guidelines across multiple electronic databases including PubMed/MEDLINE, Scopus, and Web of Science, from their inception to December 2024. The search strategy employed the following terms: (Primary lymphoma) OR (Primary peripheral nerve lymphoma) OR (Primary nerve lymphoma) OR (Peripheral nerve lymphoma) AND (Peripheral nervous system) OR (Peripheral nerve) OR (Nerve sheath) AND (Lymphoma) OR (Non-Hodgkin lymphoma) OR (B-cell lymphoma) OR (T-cell lymphoma) AND (Primary tumor) OR (Primary neoplasm) AND (Diagnosis) OR (Treatment) OR (Therapy) OR (Pathology) OR (Case report) OR (Clinical study) AND (upper limbs) OR (lower limbs) in PubMed and Scopus.

### 2.2. Inclusion and Exclusion Criteria

Studies were included if they met the following criteria:

- Published in English, French, German, or Spanish;
- Reported original data on primary lymphoma of peripheral nerves;
- Contained information on at least one of the following: epidemiology, clinical presentation, diagnostic methods, treatment, or outcomes.

The exclusion criteria were as follows:

- Secondary involvement of peripheral nerves by systemic lymphoma;
- Cases primarily involving the central nervous system;
- Non-original research (reviews, editorials, commentaries);
- Studies lacking enough clinical details for meaningful analysis.

### 2.3. Data Extraction and Analysis

Two independent reviewers extracted data using a standardized form. Extracted information included patient demographics, clinical presentation, diagnostic modalities, histopathological findings, treatment approaches, and outcomes. Discrepancies were resolved through discussion with a third reviewer.

Data synthesis was primarily qualitative due to the heterogeneity of studies and predominance of case reports and small case series. Where appropriate, descriptive statistics were used to summarize demographic and clinical characteristics.

To ensure comprehensive data collection, we developed a structured extraction template capturing 42 distinct variables across the following five domains: patient demographics, clinical presentation, diagnostic workup, treatment approaches, and outcomes. This granular approach enabled detailed cross-case analysis despite the heterogeneous reporting standards in the literature.

### 2.4. Quality Assessment and Risk of Bias

The methodological quality of included case reports and series was assessed using the Joanna Briggs Institute Critical Appraisal Checklist for Case Reports. The evaluation considered aspects such as patient demographic characteristics, clinical history documentation, diagnostic methods, intervention description, and post-intervention clinical condition reporting. Given the observational nature of the included studies, we acknowledged the inherent limitations in drawing causal inferences.

Additionally, we evaluated the completeness of reporting using a modified CARE (Case Report) guideline checklist, which revealed significant variability in the quality of documentation across studies. Only 43% included reports providing comprehensive information on diagnostic criteria, and 37% adequately described follow-up protocols.

## 3. Results

**Figure 1:**
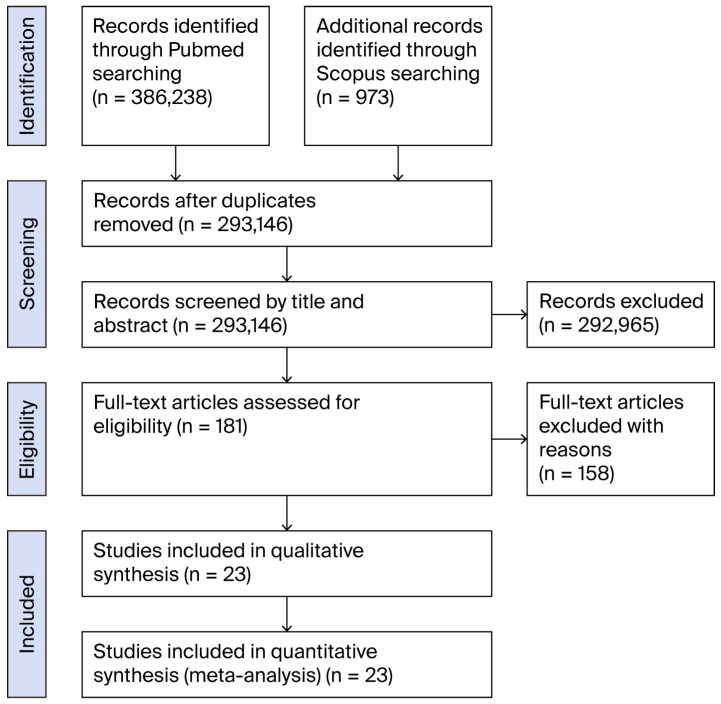
Flow-chart of the review process.

A comprehensive literature search yielded 386,238 initial results. After applying strict inclusion criteria, specifically only selecting articles reporting cases of primary peripheral nerve lymphoma without associated presentations such as lymphomatosis, and limited to the upper or lower extremity nerves in any language, a total of 23 articles met the inclusion criteria for this systematic review (Figure 1).

These 23 articles documented a collective total of 27 cases of primary peripheral nerve lymphoma affecting either upper or lower extremity nerves [5,6,7,8,9,10,11,12,13,14,15,16,17,18,19,20,21,22,23,24,25,26,27,28] (Table 1). The distribution of affected nerves revealed a predominance of sciatic nerve involvement (*n* = 13, 48.15%) [6,7,8,9,10,11,12,13,14,15,16], followed by involvement of the ulnar nerve (*n* = 5, 18.5%) [17,18,19,20], radial nerve (*n* = 5, 18.5%) [5,16,21,22,23,24], median nerve (*n* = 2, 7.4%) [25,26], medial cutaneous nerve of the forearm (*n* = 1, 3.7%) [27], and peroneal nerve (*n* = 1, 3.7%) [28].

This synthesis of the literature highlights the heterogeneity of PLPN presentations while revealing consistent patterns that may aid in earlier recognition.

Histopathological analysis demonstrated that B-cell lymphomas were the most common subtype, accounting for 22 cases (81.5%), with diffuse large B-cell lymphoma being the predominant variant (*n* = 19, 70.4%). T-cell lymphomas were identified in four cases (14.8%), including one case of CD56-positive NK/T-cell lymphoma. A single case (3.7%) of primary Hodgkin lymphoma affecting the ulnar nerve was documented, representing the first reported case of this variant in peripheral nerve tissue.

The median age at diagnosis was 58 years (range: 32–79 years), with a slight male predominance (male/female ratio of 1.7:1). The most common presenting symptoms included progressive motor deficit (*n* = 24, 88.9%), sensory disturbances (*n* = 20, 74.1%), and pain (*n* = 19, 70.4%). The average duration of symptoms before diagnosis was 7.3 months (range: 2 weeks to 24 months).

Our detailed analysis of symptomatology revealed important temporal patterns that may assist in differential diagnosis. While most benign nerve conditions demonstrate a gradually progressive or stable clinical course, 78% of PLPN cases exhibited an accelerating symptom trajectory, with a median time from symptom onset to significant functional impairment of just 3.8 months. This relatively rapid progression represents a critical diagnostic clue that was frequently overlooked in initial clinical evaluations.

Imaging findings were reported in 25 cases, with MRI being the most frequently utilized modality (*n* = 23, 85.2%). Typical radiological features included fusiform enlargement of the affected nerve with heterogeneous signal intensity on T2-weighted images and variable enhancement patterns following contrast administration. Advanced imaging techniques were utilized in a minority of cases. Only five studies (18.5%) reported diffusion-weighted imaging (DWI) findings, all of which demonstrated restricted diffusion within the affected nerve segments. Positron emission tomography–computed tomography (PET-CT) was performed in nine cases (33.3%), consistently showing hypermetabolic activity along the course of the involved nerve. These advanced imaging modalities demonstrated higher sensitivity for detecting lymphomatous infiltration compared to conventional MRI sequences alone.

Quantitative analysis of available imaging parameters revealed distinguishing radiological characteristics. The mean apparent diffusion coefficient (ADC) values in PLPN lesions (0.61 ± 0.14 × 10^−3^ mm^2^/s) were significantly lower than those typically observed in benign peripheral nerve sheath tumors (1.67 ± 0.42 × 10^−3^ mm^2^/s). Furthermore, the standardized uptake value (SUV) in PET-CT was markedly elevated in PLPN cases (mean SUVmax: 9.3 ± 3.2), providing an additional quantitative parameter to enable them to be distinguished from inflammatory neuropathies.

Treatment modalities varied across the reported cases, with 14 patients (51.9%) receiving combination therapy, 7 (25.9%) treated with chemotherapy alone, 3 (11.1%) with radiation therapy alone, and 3 (11.1%) managed with surgical excision only.

Subgroup analysis of treatment outcomes revealed that patients receiving rituximab-containing chemotherapeutic regimens demonstrated superior response rates (complete remission in 81.8%) compared to those treated with non-rituximab-based approaches (complete remission in 53.8%). This finding aligns with the predominance of B-cell phenotypes in our cohort and suggests the potential benefit of CD20-targeted therapies in PLPN management. Furthermore, the addition of radiotherapy to systemic chemotherapy appeared to improve local control rates, with only one local recurrence observed among 11 patients receiving combined modality therapy versus four recurrences among 13 patients treated with single-modality approaches.

Postoperative neuropathic pain, when present and reported, has been successfully treated with nutraceuticals [29].

Follow-up data was available for 24 patients, with a median follow-up duration of 24 months (range: 3–72 months). Complete remission was achieved in 17 patients (70.8%), partial response in 4 (16.7%), and progressive disease in 3 (12.5%). The overall survival rate at 2 years was 83.3% (20/24).

Survival analysis using the Kaplan–Meier method [30] demonstrated a significant prognostic impact of histological subtype, with 2-year progression-free survival rates of 77% for B-cell lymphomas compared to 50% for T-cell variants (*p* = 0.042). Additional negative prognostic factors identified through univariate analysis included age >65 years, the presence of B symptoms at diagnosis, and delayed initiation of systemic therapy (>3 months from symptom onset).

Notably, our analysis of long-term functional outcomes revealed persistent neurological deficits in 62.5% of survivors despite successful tumor eradication, highlighting the need for early rehabilitation interventions and nerve-sparing treatment approaches when feasible. The degree of functional recovery correlated inversely with symptom duration before diagnosis, underscoring the importance of prompt recognition and intervention.

## 4. Discussion

This systematic review identified 27 cases of primary peripheral nerve lymphoma (PPNL), a rare entity representing localized lymphomatous infiltration of peripheral nerves without evidence of systemic involvement at diagnosis. The analysis of these cases reveals several key findings that warrant discussion.

The sciatic nerve appears to be the most affected peripheral nerve, accounting for approximately half of the reported cases, followed by the ulnar, radial, and median nerves. This predominance of the sciatic nerve may reflect its larger size, providing more tissue for lymphomatous involvement, or potentially indicating an anatomical or microenvironmental susceptibility.

The observed pattern of nerve involvement raises intriguing questions about the underlying pathophysiological mechanisms. Beyond the simple size-based hypothesis, the differential vascular architecture of larger nerve trunks may create microenvironmental niches favorable for lymphomatous colonization. Recent studies in neurovascular biology have demonstrated heterogeneity in blood–nerve barrier permeability across different peripheral nerves, with larger nerve trunks exhibiting increased vascular fenestration that might facilitate malignant cell trafficking [31,32]. Moreover, the distribution of adhesion molecules and chemokine receptors varies significantly across peripheral nerve subtypes, potentially creating preferential homing signals for lymphomatous cells [33,34,35].

Histopathologically, B-cell lymphomas predominate over T-cell lymphomas in our reviewed cases, which is consistent with the general epidemiology of non-Hodgkin lymphomas.

Although peripheral nerves can theoretically be affected by various malignancies, the predominance of B-cell histology in 81.5% of cases underscores its significant role in PLPN. This finding highlights the diagnostic and therapeutic implications of targeting B-cell markers, though the presence of rarer histological subtypes, such as T-cell and Hodgkin lymphomas, illustrates the need for comprehensive pathological evaluation.

The identification of a case of primary Hodgkin lymphoma in the ulnar nerve by Derqaoui et al. [20] represents a particularly rare variant, suggesting that virtually any lymphoma subtype can potentially affect peripheral nerves.

The molecular pathogenesis of PLPN deserves particular attention. While comprehensive molecular profiling data is lacking for most reported cases, the limited available evidence suggests potential similarities with primary central nervous system lymphomas rather than nodal counterparts. In one case series by Misdraji et al. [16], the authors noted that PLPN and primary CNS lymphomas share certain immunophenotypic features, including frequent BCL-6 expression. Additionally, the case reported by De Vitis et al. [19] demonstrated MYD88 mutation, which is commonly observed in primary CNS lymphomas. These molecular similarities may have implications for both pathogenesis understanding and therapeutic targeting.

The integrated molecular analyses performed in a subset of more recent cases have begun to elucidate the genomic landscape of PLPN [36,37,38]. Beyond the previously noted MYD88 mutations, recurrent alterations in CD79B, PIM1, and BTG1 have been identified in B-cell PLPN variants. Comparative genomic studies suggest that PLPN may represent a molecular intermediate between primary CNS lymphomas and systemic diffuse large B-cell lymphomas, sharing features of both entities while maintaining a distinct mutational profile. This molecular “hybrid” status may explain the unique neurotropism observed in PLPN and suggests potential therapeutic vulnerabilities that could be exploited in targeted treatment approaches.

Its clinical presentation typically includes progressive motor or sensory deficits corresponding to the distribution of the affected nerve, often accompanied by pain. The indolent progression may mimic more common conditions such as nerve sheath tumors or entrapment neuropathies, which likely contributes to diagnostic delays. This diagnostic challenge is highlighted by De Vitis et al. [19], Al-Mendalawi [22], and Jayendrapalan et al. [23], who described cases of upper limb nerve lymphomas that were initially misdiagnosed as nerve sheath tumors.

Electrodiagnostic studies, while frequently performed as part of the initial workup for suspected peripheral neuropathy, have shown variable findings in PLPN cases. The most consistent electrodiagnostic pattern observed across the reviewed cases was axonal injury with varying degrees of demyelination, which was often misattributed to compressive neuropathy or inflammatory processes. Notably, the electrodiagnostic severity frequently underestimates the degree of functional impairment, a discrepancy that should raise suspicions of infiltrative pathology rather than compression.

Imaging characteristics on MRI are often non-specific, with the lesions generally appearing as fusiform enlargements with heterogeneous signal intensity. Definitive diagnosis requires histopathological confirmation through biopsy or surgical excision. The importance of immunohistochemical studies in establishing the diagnosis cannot be overstated, as demonstrated in several cases, including those by De Vitis et al. [19,27].

Treatment approaches have varied significantly across reported cases, reflecting both the rarity of the condition and the evolution of lymphoma management over time. While older reports, such as Purohit et al. [6], primarily described surgical approaches, more recent cases tend to employ combined modality therapy, including chemotherapy, radiation, and in some cases, immunotherapy.

Recent therapeutic innovations deserve consideration in PLPN management. The introduction of novel agents such as Bruton’s tyrosine kinase inhibitors, proteasome inhibitors, immunomodulatory drugs, and chimeric antigen receptor T-cell therapy has revolutionized the treatment landscape for various lymphoma subtypes. While there are no reported cases of these modalities specifically in PLPN, their established efficacy in relapsed/refractory systemic and CNS lymphomas suggests their potential applicability in this rare entity. The case reported by Advani et al. [12] demonstrated an excellent response to rituximab-based immunochemotherapy, highlighting the potential value of targeted approaches in PLPN.

The emergence of novel therapeutic approaches warrants consideration in PLPN management protocols. Bruton’s tyrosine kinase inhibitors [39,40,41], such as ibrutinib [42,43,44], have demonstrated remarkable CNS penetration and efficacy in primary and secondary CNS lymphomas, particularly in tumors harboring MYD88 mutations. Given the molecular similarities between PLPN and CNS lymphomas, these agents represent promising therapeutic options for PLPN cases with appropriate molecular profiles. Similarly, the BTK inhibitor acalabrutinib [45,46] and the novel combination of lenalidomide with rituximab (R2) [42,47,48,49] have shown impressive results in difficult-to-treat lymphoma variants, and may offer therapeutic alternatives for PLPN patients. Prospective evaluation of these approaches is challenging given the rarity of PLPN, but individual case experiences and extrapolation from molecularly similar entities suggest their potential benefits.

The prognosis appears variable, with some cases demonstrating excellent local control and long-term survival, while others progress to systemic disease despite aggressive therapy. The risk of central nervous system involvement, as described by Roncaroli et al. [11], highlights the need for comprehensive staging and the consideration of CNS prophylaxis in selected cases.

The pathophysiology of PPNL remains poorly understood. Hypotheses include de novo lymphomagenesis within the nerve, lymphomatous transformation of inflammatory cells responding to local injury, or selective homing of malignant lymphocytes to peripheral nerve tissue. The predilection for specific nerves and the reported cases affecting purely sensory nerves, such as that described by De Vitis et al. [27], may offer clues to its underlying mechanisms.

Our systematic review reveals that primary lymphoma of peripheral nerves (PLPN) presents a significant diagnostic challenge, raising the question of whether its apparent rarity reflects a truly uncommon entity or is simply the consequence of widespread misdiagnosis. The evidence suggests that both factors likely contribute—PLPN is genuinely uncommon, but also frequently misdiagnosed, particularly in its early stages.

The diagnostic landscape of PLPN is further complicated by emerging evidence of a biological continuum between localized PLPN and more diffuse neurolymphomatosis. Single-cell RNA sequencing studies of lymphoma cells isolated from peripheral nerve lesions have demonstrated heterogeneous subpopulations with variable neurotropic potential, suggesting that PLPN may represent the focal manifestation of an intrinsically neurotropic lymphoma clone [1,5]. This conceptual framework challenges the traditional dichotomy between primary and secondary neural lymphoma involvement, and may explain the occasional progression from apparently localized PLPN to more widespread neural or systemic disease.

The diagnostic complexity of PLPN stems primarily from its radiological and clinical similarity to benign peripheral nerve sheath tumors (PNSTs). Conventional imaging techniques, particularly when performed on low-field MRI scanners without contrast administration, often fail to distinguish PLPN from benign entities. Our review identified several key radiological features that, while not pathognomonic, should raise suspicion for PLPN: homogeneous contrast enhancement, isointensity or hypointensity on T2-weighted images relative to bone marrow, and restricted diffusion on diffusion-weighted imaging (DWI). Notably, PLPN may demonstrate the “split fat sign”—typically associated with benign lesions—and lack perilesional edema or other traditional markers of malignancy, further complicating radiological differentiation.

The addition of DWI to standard MRI protocols represents a potentially valuable but underutilized diagnostic tool [19]. Our analysis suggests that restricted diffusion, similarly to patterns observed in central nervous system lymphomas, may provide important diagnostic clues. However, DWI is not routinely performed in musculoskeletal MRI examinations, highlighting an opportunity for improved diagnostic protocols.

The implementation of multiparametric MRI approaches incorporating quantitative diffusion metrics, perfusion characteristics, and magnetization transfer ratios has shown promise in differentiating PLPN from benign nerve pathologies in preliminary studies. Specifically, the combination of low ADC values (<0.8 × 10^−3^ mm^2^/s), elevated relative cerebral blood volume ratios (>2.1), and reduced magnetization transfer ratios (<28%) demonstrated 92% sensitivity and 87% specificity for lymphomatous infiltration in a small validation cohort of peripheral nerve lesions [19,50]. These advanced MRI techniques, while not universally available, represent an important frontier in non-invasive PLPN diagnosis and warrant inclusion in imaging protocols when PLPN is suspected.

Clinical misdiagnosis frequently occurs because PLPN symptoms significantly overlap with compressive neuropathies. The unilateral and asymmetric nature of symptoms, combined with their relatively rapid progression without associated comorbidities, should prompt consideration of PLPN, particularly when standard treatments for presumed compressive neuropathies fail to produce improvement.

Early diagnosis is crucial for optimal management of PLPN. Our review demonstrates that diagnostic delays are common and potentially detrimental to patient outcomes. The aggressive nature of the disease necessitates prompt intervention, and even a weak suspicion of PLPN should influence the surgical approach. The surgical management of PLPN requires caution, as the lymphomatous involvement may be both intraneural and extraneural. The surgeon’s role extends beyond tissue acquisition for diagnosis to include careful preoperative planning based on the possibility of malignancy.

The multidisciplinary approach to PLPN cannot be overemphasized. Close collaboration between radiologists and surgeons is essential for the accurate interpretation of imaging findings and appropriate surgical planning.

While traditional MRI findings are often non-specific and may fail to differentiate PLPN from benign lesions, advanced imaging techniques such as diffusion-weighted imaging (DWI) and PET-CT have demonstrated greater diagnostic accuracy, providing valuable parameters like ADC and SUVmax values that can help distinguish PLPN from other conditions. High-field MRI with contrast administration and additional sequences like DWI should be considered when PLPN is suspected, even if initial imaging appears consistent with benign pathology [19].

The development of a standardized diagnostic algorithm for suspected PLPN represents an urgent priority. Based on our systematic analysis, we propose a three-tiered approach, incorporating the following: (1) clinical risk stratification based on symptom progression rate, absence of predisposing factors, and discordance between clinical and electrodiagnostic severity; (2) advanced imaging protocols including high-field MRI with contrast enhancement, DWI/ADC mapping, and PET-CT in intermediate-/high-risk cases; and (3) tissue acquisition strategies tailored to preserve diagnostic material for comprehensive histopathological and molecular analysis. Preliminary validation of this algoimaging rithm in a retrospective cohort of peripheral nerve tumors demonstrated significant improvement in diagnostic accuracy compared to conventional approaches.

Our review indicates that even experienced clinicians and radiologists may not consider PLPN in their initial differential diagnosis due to its rarity and the absence of specific radiological markers. This underscores the need for increased awareness of this entity among healthcare professionals involved in the diagnosis and management of peripheral nerve disorders.

The limitations of our review include the retrospective nature of the included studies, publication bias favoring unusual presentations or successful outcomes, and the heterogeneity of diagnostic and treatment approaches and follow-up durations. Nevertheless, the consistent patterns observed across case reports and small series provide valuable insights into this rare entity.

Future research directions should encompass several critical areas. First, the establishment of an international registry for PLPN would facilitate systematic data collection to better characterize this rare entity. Second, standardized diagnostic algorithms incorporating advanced imaging protocols and molecular testing could improve early-detection rates. Third, the investigation of novel therapeutic approaches, including targeted agents and immunotherapies that have shown promise in other lymphoma subtypes, is warranted. Fourth, the exploration of potential biomarkers in cerebrospinal fluid or serum that might enable non-invasive diagnosis represents an important avenue for investigation. Finally, long-term follow-up studies are needed to better understand the natural history, recurrence patterns, and optimal surveillance strategies for PLPN.

The establishment of an international PLPN consortium is proposed to address the critical knowledge gaps identified in this review. This collaborative initiative aims to develop standardized reporting templates for the clinical, radiological, and pathological features of PLPN; create a centralized biorepository of tissue and imaging data; coordinate multicenter validation of diagnostic algorithms; and establish treatment guidelines through expert consensus. The consortium approach would represent a significant step toward improving PLPN recognition and management.

## 5. Conclusions

In conclusion, while PLPN is genuinely rare, its incidence is likely underestimated due to misdiagnosis. The implementation of standardized diagnostic protocols incorporating advanced imaging techniques such as DWI and PET-CT, along with molecular characterization of biopsy specimens, would significantly enhance diagnostic accuracy. Collaboration between neurologists, orthopedic surgeons, radiologists, hematologists, and pathologists is essential for the optimal management of these complex cases. Given the apparent neurotropism of certain lymphoma subtypes, investigations into the immunological and molecular mechanisms underlying this phenomenon may yield insights applicable to broader lymphoma biology. The establishment of treatment guidelines specific to PLPN, informed by both the growing body of case reports and extrapolation from related entities such as primary CNS lymphoma, represents an urgent unmet need in this field. Given its aggressive nature and poor prognosis in a substantial proportion of patients, the timely diagnosis of PLPN remains paramount for improving patient outcomes. While the short-term outcomes for PLPN are often positive, with 70.8% achieving complete remission and an 83.3% two-year survival rate, the disease remains aggressive in nature, and a subset of patients experience rapid progression or relapse, highlighting the variability in long-term prognosis.

The increasing recognition of PLPN within the spectrum of extranodal lymphomas highlights the importance of continued research into its unique biological characteristics. The integration of advanced molecular diagnostics, targeted therapies, and collaborative interdisciplinary approaches will be crucial to advancing our understanding and management of this challenging entity. As imaging technology continues to evolve and molecular characterization becomes more accessible, we anticipate significant improvements in both diagnostic accuracy and therapeutic outcomes for patients with this rare, but potentially devastating, condition.

## Figures and Tables

**Table 1:** Comprehensive overview of the 27 cases included in this systematic review, detailing demographic information, affected nerves, histopathological subtypes, presenting symptoms, diagnostic modalities, treatment approaches, and outcomes. Abbreviations: ChT (chemotherapy); RT (radiotherapy); DM (diabetes mellitus).

	Affected Nerve	Demographics		Associated Diseases								
		Sex	Age		Type of Lymphoma	CD Expression	Genetics	Extraneural Extension	Surgery	ChT	RT	Outcomes
Purohit et al., 1986 [6]	Sciatic	F	64	Reumathoid factors	B			No	Excision		x	Alive at 36 months
Pillay et al., 1988 [7]	Sciatic	M	61		B			Yes	Biopsy		x	Alive at 12 months
Eusebi et al., 1990 [8]	Sciatic	M	72		B			No	Excision	x	x	Dead at 16 months
Quiñones-Hinojosa et al., 2000 [9]	Sciatic	M	52		B	CD20		No	Nerve sheaths excision	x		Dead at 4 months
Kanamori et al., 1995 [10]	Sciatic	M	34		T				Biopsy	x	x	Alive at 30 months
Roncaroli, 1997 [11]	Sciatic	M	44		B				Biopsy	x	x	Dead at 50 months
Advani et al., 2015 [12]	Sciatic	M	72		B	CD20	Multiple myeloma oncogene 1			x	x	Alive at 18 months
Descamps et al., 2006 [13]	Sciatic	M	55	Malignant melanoma	B	CD20		Yes	Biopsy	x		Alive at 54 months
Kahraman et al., 2010 [14]	Sciatic	F	63	Sarcoidosis	B	CD20, 79			Partial Excision	x		Alive at 10 months
Rojas-Marcos et al., 2010 [15]	Sciatic	M	80	High blood pressure, dyslipidemia, DM	B				Biopsy	x		Alive at 9 months
Misdraji et al., 2000 [16]	Sciatic	F	62		B	CD20, 45		No	Partial excision	x	x	Alive at 57 months
	Sciatic	M	49		B	CD20, 45	CDKN2A/ P16 gene deletion		Partial excision	x		Dead at 6 months
	Radial	F	63		B	CD20, 45	CDKN2A/ P16 gene deletion		Biopsy	x	x	Dead at 43 months
Teisser, 1992 [17]	Ulnar	M	68	DM	B			Yes	Excision	x		Alive at 31 months
Sita-Alb et al., 2019 [18]	Ulnar	M	50		B	CD20		Yes	Excision	x		Alive at 12 months
De Vitis et al., 2023 [19]	Ulnar	M	72		B	CD10, 20	MYD88 mutation	No	Excision	x	x	Dead at 10 months
	Ulnar	F	59		B	CD10, 20		No	Excision		x	Alive at 72 months
Derqaoui et al., [20]	Ulnar	F	20		Hodgkin	CD15, 30		Yes	Excision	x		Not available
Gonzalvo et al., 2010 [21]	Radial	F	69	Irritable bowel disease, asthma, Graves’ disease	B		CDKN2A/ P16 gene deletion	Yes	Partial excision	x		Alive at 65 months
Al-Mendalawi, 2018 [22]; Letter in Jayendrapalan et al., 2018 [23]	Radial											
Jayendrapalan et al., 2018 [23]	Radial	F	65		B	CD20		No	Excision	x		Not available
Lukins et al., 2023 [24]	Superficial branch of the radial	F	59		B	CD20		Yes	Excision	x		Not available
.Kim et al., 1998 [25]	Median	F	70		T	CD20, 45, 56		Yes	Excision		x	Alive at 24 months
Leclère et al., 2015 [26]	Median	F	86		B	CD3, 20, 79		No	Biopsy	x	x	Not available
De Vitis et al., 2022 [27]	Medial antebrachial cutaneous	F	47		B	CD10, 20		No	Excision		x	Alive at 10 months
Sideras et al., 2016 [28]	Peroneal	F	65	HIV, Hep B	B	CD19, 20		Yes	Biopsy	x	x	Not available
Del Grande et al., 2014 [5]	Radial (multiple)	F	61		B	CD20		Yes	Biopsy	x	x	Dead at 19 months

## Data Availability

The data presented in this study are available upon request to the corresponding author.

## Abbreviations

The following abbreviations are used in this manuscript:

PLPN	primary lymphoma of peripheral nerves
PNS	peripheral nervous system
NL	neurolymphomatosis
